# Fibrillary Glomerulonephritis in a Patient With Sjögren's Syndrome and Primary Biliary Cholangitis: A Case Report and Literature Review

**DOI:** 10.7759/cureus.75701

**Published:** 2024-12-14

**Authors:** Rita Afonso, Roberto Calças Marques, Helena Viana, Ana Cabrita, Ana Paula Silva

**Affiliations:** 1 Nephrology Department, Unidade Local de Saúde (ULS) Algarve - Hospital de Faro, Faro, PRT; 2 Nephrology Department, Unidade Local de Saúde (ULS) São José - Hospital Curry Cabral, Lisbon, PRT

**Keywords:** corticosteroids, fibrillary glomerulonephritis, non-amyloid deposits, primary biliary cholangitis, sjögren's syndrome

## Abstract

Fibrillary glomerulonephritis (FGN) is a rare glomerular disease characterized by the deposition of fibrils within the mesangium and glomerular basement membrane. Most cases are idiopathic, but it can be linked to autoimmune diseases, neoplasms, and infections. There is limited evidence on the best treatment approach, and many patients progress to end-stage kidney disease. The authors present a case of FGN in a patient with Sjögren's syndrome (SS) and primary biliary cholangitis (PBC), highlighting a successful clinical outcome.

## Introduction

Fibrillary glomerulonephritis (FGN) is a rare glomerular disease found in less than 1% of native kidney biopsies [1] and typically presents with nephrotic-range proteinuria, hypertension, microscopic hematuria, and renal failure [2]. It is more common in women, with the mean age at diagnosis ranging from 49 to 60 years [3]. Pathologically, FGN is defined by the ultrastructural finding of organized, randomly oriented, non-branching fibrils with a mean diameter of approximately 20 nm, located in the mesangium and/or along the glomerular basement membrane [4]. Glomerular deposits in FGN usually stain negative with Congo red and show positive immunofluorescence for IgG, complement, kappa, and lambda light chains, consistent with immune complex-mediated glomerulonephritis (GN), in which IgG deposits are polymerized into fibrils [3,5]. Recently, a highly sensitive and specific tissue marker for FGN, DnaJ homolog subfamily B member 9 (DNAJB9), was identified and may act as an autoantigen in this disease [6]. Most cases of FGN are idiopathic, though it has been associated with hepatitis C virus (HCV) infection, monoclonal gammopathies, malignancies, and various autoimmune disorders [3].

FGN carries a poor prognosis, with limited data to suggest optimal therapy. Nearly 50% of patients progress to end-stage renal disease within four years of diagnosis [7].

We describe a case of biopsy-proven FGN in a patient with Sjögren's syndrome (SS) and primary biliary cholangitis (PBC).

## Case presentation

History and physical examination

A 79-year-old woman with a past medical history of hypertension and PBC for two years, both well controlled with antihypertensive agents and ursodeoxycholic acid, respectively, presented to the emergency department with a several-week history of generalized fatigue, anorexia, and gradually worsening swelling of the lower extremities. The patient also reported oliguria for the past week, without foamy urine or hematuria. There was no known history of renal disease. Along with this, she had experienced dry mouth and dry eyes for 10 years. She denied shortness of breath, rash, joint pain, hemoptysis, pruritus, fever, or recent illnesses.

On admission, the patient was hypertensive (blood pressure 140/86 mmHg), with pale skin and bilateral 4+ pitting edema of the lower extremities. The rest of the physical examination was unremarkable.

Hospital course

**Figure 1:**
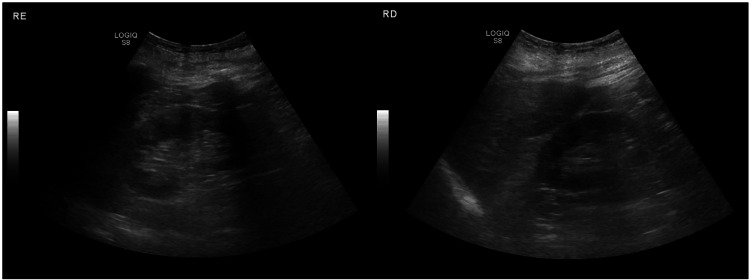
Renal ultrasonography Mild loss of corticomedullary differentiation in both kidneys, with slightly increased cortical echogenicity and prominence of the medullary pyramids in the left kidney (left figure). Renal size and parenchymal thickness were preserved bilaterally.

An initial laboratory workup revealed normocytic normochromic anemia (hemoglobin 72 g/L) with mild thrombocytopenia (platelet count 135×10^9^/L), normal serum liver tests, and acute kidney injury Kidney Disease: Improving Global Outcomes (KDIGO) stage 3 (creatinine 5.9 mg/dL and blood urea nitrogen 114 mg/dL) associated with hyperkalemia (potassium 6 mmol/L) and metabolic acidosis. The lipid profile was normal, but hypoalbuminemia was present (albumin 1.8 g/dL). Urinalysis revealed protein 4+ and erythrocytes 2+, with a 24-hour urine proteinuria of 20,000 mg. Renal ultrasonography showed mild loss of corticomedullary differentiation in both kidneys, with slightly increased cortical echogenicity and prominence of the medullary pyramids in the left kidney. Renal size and parenchymal thickness were preserved bilaterally (Figure 1).

Additional laboratory investigations were positive for anti-nuclear antibodies (ANA) with a mixed pattern, anti-Ro/SSA, anti-La/SSB, and anti-mitochondrial antibodies (AMA). Anti-double-stranded DNA (anti-dsDNA), anti-neutrophil cytoplasmic antibodies (ANCA), circulating cryoglobulins, human immunodeficiency virus (HIV), hepatitis B, and hepatitis C tests were negative. Serum complement C3 levels were reduced (C3 0.48 g/L; normal range 0.79-1.52), while serum C4 levels were normal (C4 0.163g/L; normal range 0.160-0.380). Discrete elevation of IgA levels was noted, but no hypergammaglobulinemia, and serum immunofixation did not reveal any monoclonal immunoglobulin (Table 1).

**Table 1:** Laboratory data WBC: white blood cell; ALT: alanine aminotransferase; AST: aspartate aminotransferase; ANA: anti-nuclear antibodies; anti-dsDNA: anti-double-stranded DNA; anti-Sm: anti-Smith antibody; AMA: anti-mitochondrial antibodies; ANCA: anti-neutrophil cytoplasmic antibodies; HIV: human immunodeficiency virus

Parameters	Patient values	Reference range
Hemoglobin, g/L	72	115-155
Absolute WBC count, ×10^9^/L	5.0	4.0-10.0
Absolute platelet count, ×10^9^/L	135	150-400
ALT, UI/L	<6	<55
AST, UI/L	14	5-34
Creatinine, mg/dL	5.9	0.6-1.1
Blood urea nitrogen, mg/dL	114	9.8-20.1
Potassium, mmol/L	6.0	3.6-5.1
Albumin, g/dL	1.8	3.4-4.8
Total cholesterol, mg/dL	116	<200
Ferritin, ng/mL	284	20-300
Transferrin saturation, %	33	20-55
Serum complement C3 level, g/L	0.48	0.79-1.52
Serum complement C4 level, g/L	0.163	0.160-0.380
Immunoglobulin A, g/L	6.83	0.82-4.53
Serum protein electrophoresis	No hypergammaglobulinemia	
Serum immunofixation	No monoclonal immunoglobulin	
ANA	Positive, with a speckled pattern and more granular cytoplasmatic staining	
Anti-dsDNA	Negative	
Anti-Sm	Negative	
Anti-Ro/SSA	Positive	
Anti-La/SSB	Positive	
AMA	Positive	
ANCA	Negative	
Circulating cryoglobulins	Negative	
HIV	Negative	
Hepatitis B	Negative	
Hepatitis C	Negative	
C-reactive protein, mg/L	6	
Urinalysis protein	4+	
Urinalysis red blood cell	2+	
24-hour urine proteinuria (mg/24 h)	20000	<300

A thoraco-abdominopelvic computed tomography scan was performed to exclude neoplastic etiology due to the presence of constitutional symptoms and as part of the investigation of nephrotic-range proteinuria. No abnormalities were found.

**Figure 2:**
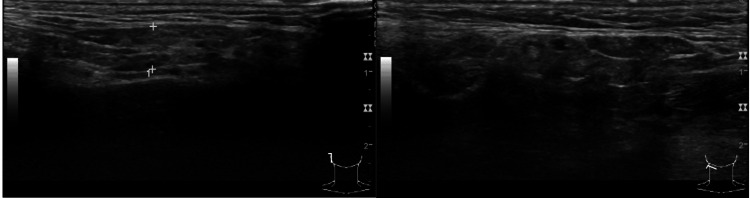
Salivary gland ultrasonography (parotid gland) There is a dimensional reduction of the parotid (left image) and submandibular glands (right image), which present a heterogeneous parenchyma, predominantly hypoechoic, with some rounded millimeter images that are more hypoechoic, translating a multicystic appearance. These aspects suggest atrophy of the glands due to a chronic inflammatory process.

Given the presumed diagnosis of SS (based on ocular and oral symptoms, as well as positive serum antibodies to SSA and SSB antigens), a salivary gland ultrasonography was performed, revealing bilateral submandibular and parotid gland atrophy (Figure 2).

**Figure 3:**
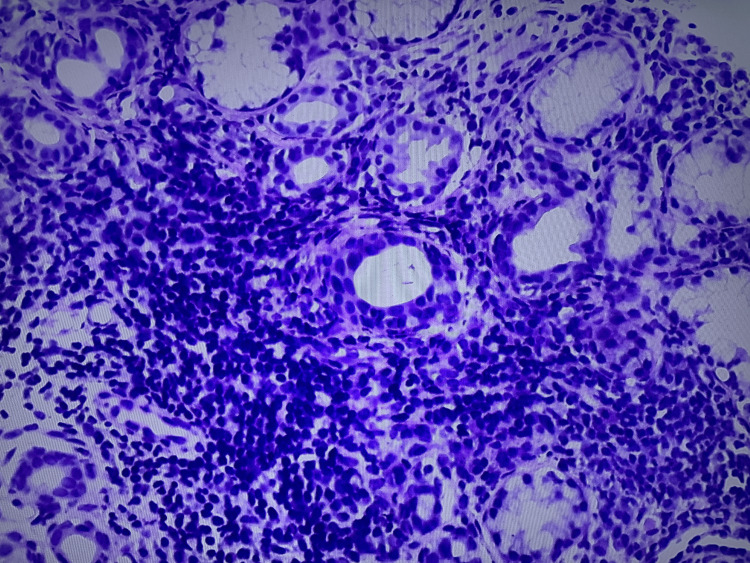
Biopsy of the minor salivary glands Marked sialadenitis with lymphocytic infiltrates (hematoxylin and eosin stain; original magnification, ×200).

Biopsy of the minor salivary glands confirmed the initial suspicion, showing marked sialadenitis with lymphocytic infiltrates (Figure 3).

**Figure 4:**
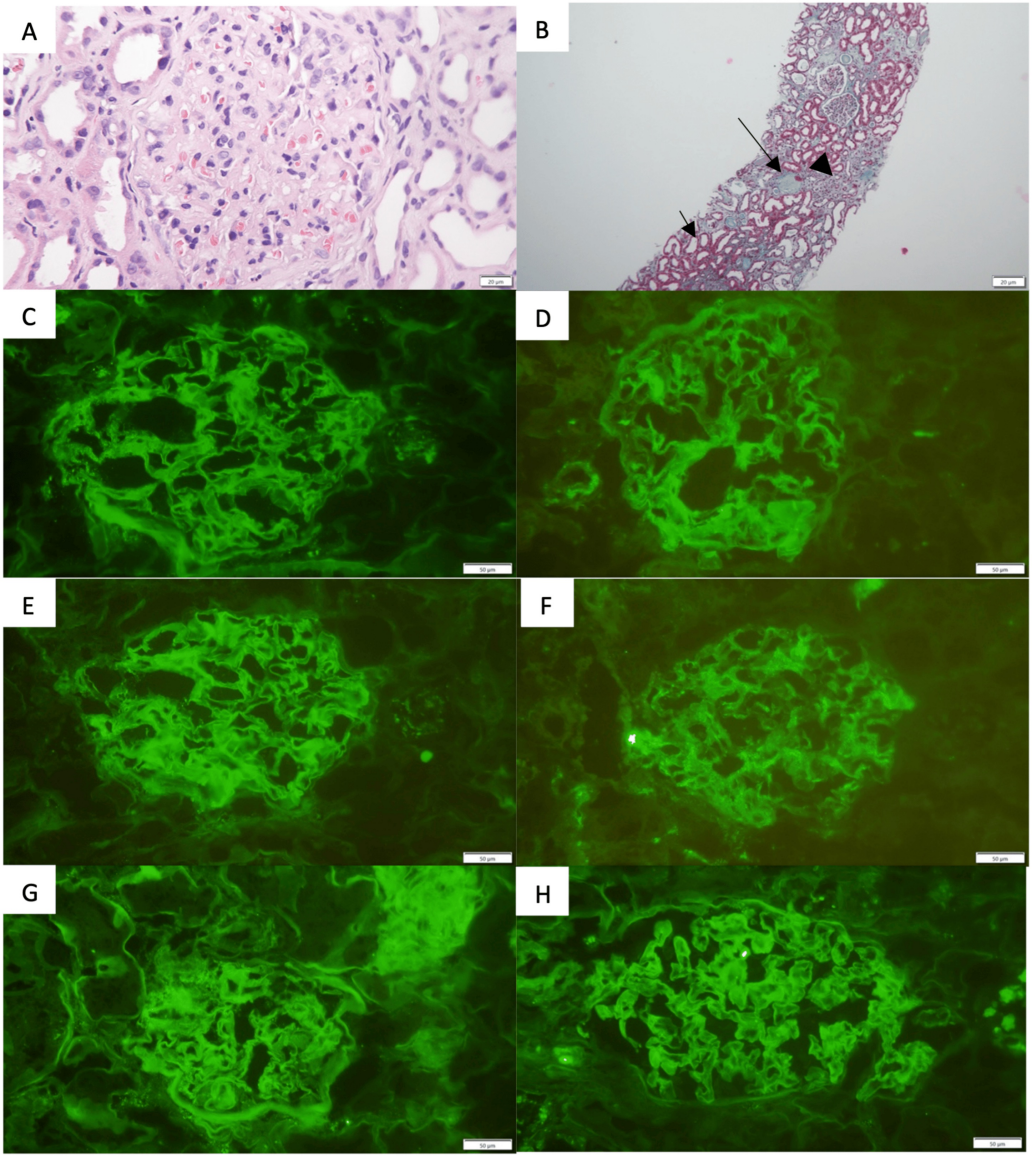
Kidney biopsy findings (A) The glomerulus shows thickening of the capillary wall and heterogeneous mesangial enlargement (hematoxylin and eosin stain; original magnification, ×400). (B) Glomerulus with global sclerosis (large arrow), extensive acute tubular injury (small arrow), and foci of interstitial fibrosis with moderate mononuclear inflammatory infiltrates in fibrosis areas (Masson's trichrome stain; original magnification, ×400). Glomerular deposits stain brightly for (C) IgG ++ and (D) C3++ and stain moderately for (E) IgA, (F) IgM, (G) kappa, and (H) lambda (immunofluorescence images; original magnification, ×250).

A kidney biopsy was also performed to clarify the kidney involvement (Figure 4). Light microscopy revealed 17 glomeruli, seven of which exhibited global sclerosis, while the remaining glomeruli showed thickening of the capillary walls and heterogeneous mesangial enlargement due to sparse deposits. These deposits were negative for Congo red staining. Foci of interstitial fibrosis involving 25% of the cortex were present, along with moderate interstitial mononuclear inflammatory infiltrates in areas of fibrosis. Extensive acute tubular injury was also observed. The vascular compartment was preserved. Immunofluorescence showed linear mesangial-parietal deposits of IgG++, IgA+, IgM+/-, C3++, kappa+, and lambda+. Electron microscopy revealed podocyte effacement with no evidence of electron-dense deposits or microtubules. The clinical and biopsy findings were consistent with early-phase FGN and acute tubular injury.

Treatment was initiated with pulses of methylprednisolone, followed by daily oral prednisolone (0.75 mg/kg/day). Additional medical management included diuretics, an angiotensin-converting enzyme inhibitor (started close to the date of discharge, after some improvement in renal function and resolution of hyperkalemia), and ursodeoxycholic acid. Prednisolone was tapered slowly over a six-month period.

One month after discharge, the patient showed clinical improvement with regression of peripheral edema, a serum creatinine level of 1.3 mg/dL, and a reduction in 24-hour urine protein to 2,279 mg. Serum albumin levels also improved (albumin 3.4 mg/dL). This clinical improvement was maintained over time, with a creatinine level of 1.1 mg/dL and a 24-hour urine protein level of 298 mg more than a year after follow-up.

## Discussion

FGN and autoimmune disorders

Autoimmune disease appears to be the condition most commonly associated with FGN, reported in 13-30% of patients in recent series [3]. Examples of autoimmune diseases observed in patients with FGN include Crohn's disease, systemic lupus erythematosus, Graves' disease, and idiopathic thrombocytopenic purpura. A search on PubMed conducted by the authors in November 2024 using the terms "fibrillary glomerulonephritis"+"Sjögren syndrome" and "fibrillary glomerulonephritis"+"primary biliary cholangitis" revealed only three previous cases of FGN associated with SS [8,9] and one case involving a patient with primary myelofibrosis who developed PBC, autoimmune hemolytic anemia, and FGN [10]. Therefore, while the association of FGN with SS or PBC is rare, the simultaneous occurrence of FGN with both conditions is even rarer. To the best of our knowledge, we present the first case in the literature.

It is well known that the occurrence of an autoimmune disease increases the risk of developing additional autoimmune diseases in the same patient [11]. PBC is a chronic inflammatory autoimmune cholestatic liver disease that, if left untreated, progresses to end-stage biliary cirrhosis [12]. A significant portion of patients with PBC present with sicca syndrome, and some meet the criteria for classical SS. Both PBC and SS are characterized by inflammation and immune-mediated destruction of epithelial tissue, with a common autoantigen, pyruvate dehydrogenase complex-E2 (PDC-E2), identified in the bile duct and salivary gland epithelium. This has led to the hypothesis that PBC, like SS, can be considered a generalized epithelitis [11]. The similarity of the periepithelial inflammatory infiltrate in the salivary ductal epithelium and the renal tubules further supports the notion of a common immunopathogenic mechanism in SS, where renal involvement most frequently manifests as interstitial nephritis [8,13].

Glomerular involvement is less common in patients with SS and can present as rapidly progressive GN with nephritic syndrome or nephrotic syndrome, typically as a consequence of a non-epithelial disease with a secondary immune complex-mediated process [14]. Due to the heterogeneity of kidney involvement in patients with SS, kidney biopsy should be considered in all cases, as it impacts both treatment and prognosis. Membranoproliferative glomerulonephritis (MPGN) is the most frequently reported glomerular lesion; however, other glomerular diseases have also been reported, including minimal change disease, IgA nephropathy, focal segmental glomerulosclerosis, membranous nephropathy, or FGN [14], as in our clinical case.

Although electron microscopy did not show the pathognomonic finding of randomly arranged fibrils deposited in mesangial areas and along the glomerular basement membrane, possibly because the disease was at an early stage, light microscopy and immunofluorescence findings are suggestive of FGN: diffuse mesangial expansion, thickened capillary walls, negative Congo red staining, interstitial fibrosis, and prominent IgG deposits in the mesangium and along the glomerular basement membrane, along with positive staining for IgM, IgA, C3, and both kappa and lambda light chains.

The main histological differential diagnosis includes amyloidosis, which was excluded due to the negative Congo red staining. Immunotactoid glomerulopathy was also considered; however, in these cases, immunofluorescence typically shows monoclonal IgG with light chain restriction, in contrast to FGN, where polyclonal staining is observed, with kappa and lambda present in equal proportions. Furthermore, immunotactoid glomerulopathy is predominantly associated with monoclonal gammopathy and hematologic malignancies, neither of which was present in our patient [15]. Finally, cryoglobulinemic GN was excluded, as positive cryoglobulins and a membranoproliferative pattern with lobular appearance would be expected, but these findings were absent [15].

A pathophysiologic link between FGN and autoimmune disorders is plausible, considering the presence of IgG4-containing immune complexes in most cases of FGN and the recent discovery of DNAJB9 as a putative autoantigen in FGN [3]. Immunohistochemistry staining for DNAJB9 was not performed in our case, as it was not available.

Treatment

Due to the rarity of FGN and the absence of prospective randomized controlled trials, there are currently no established guidelines for its management. Based on extrapolation from evidence in other glomerulonephritides, initial treatment should involve salt restriction, optimal blood pressure control, and renin-angiotensin system blockade, the latter due to its proven renoprotective effects in reducing proteinuria and slowing kidney disease progression. Furthermore, because more than one-third of FGN patients have malignancies, monoclonal gammopathy, or autoimmune diseases, treatment of the underlying condition may prevent the progression of FGN [2]. In the specific case of SS, corticosteroids are considered first-line agents in treating tubulointerstitial nephritis and glomerular diseases, while data on the efficacy of steroid-sparing alternatives is lacking [9]. In our case, despite the patient's advanced age, massive proteinuria, and eGFR <30 ml/min/1.73 m^2^ on admission, the patient was successfully treated with systemic glucocorticoids and renin-angiotensin system blockade, without the need for additional therapies. One of the possible explanations for the favorable clinical evolution in our patient is that the diagnosis was made at an early stage [16], as evidenced by the renal biopsy findings, with <50% of the glomeruli showing global glomerulosclerosis and interstitial fibrosis present in only 25% of the sample.

Therapeutic regimens using a second agent in addition to glucocorticoids have been described in the literature, including cyclophosphamide, cyclosporine, and mycophenolate mofetil. However, these immunosuppressive approaches have shown inconsistent results in uncontrolled studies [2]. Furthermore, the use of rituximab has increased over time, given the potential autoimmune nature of FGN, particularly in patients with relatively normal baseline renal function [5]. Repository corticotrophin injections resulted in the partial remission of FGN in one patient [17], but not in another [18]. Prospective multicenter trials are needed to determine the potential benefits of these and other agents in FGN and to establish an optimal therapeutic regimen [3].

Contrary to what was observed in our patient, the prognosis of FGN is generally unfavorable, with poor response to immunosuppressive therapy. The majority of patients develop renal failure within four years after diagnosis. Renal transplantation is a viable option for patients with FGN and end-stage renal disease, but the risk of recurrence is not negligible [1].

One of the limitations of our study is that electron microscopy did not show the expected pathognomonic findings. However, after discussing the case with the experienced renal morphology unit, the findings from light microscopy and immunofluorescence, together with the clinical history, allowed us to reach the diagnosis.

## Conclusions

Despite advances in the last decade, the rarity of FGN, combined with the lack of standardized treatment, has contributed to the generally poor prognosis. Steroid therapy may be effective in FGN, even in those with impaired renal function. Controlled clinical trials are warranted to better identify patients at higher risk of unfavorable outcomes and to evaluate the efficacy of different immunosuppressive regimens.
